# Cervical Screening at Age 50–64 Years and the Risk of Cervical Cancer at Age 65 Years and Older: Population-Based Case Control Study

**DOI:** 10.1371/journal.pmed.1001585

**Published:** 2014-01-14

**Authors:** Alejandra Castañón, Rebecca Landy, Jack Cuzick, Peter Sasieni

**Affiliations:** Centre for Cancer Prevention, Wolfson Institute of Preventive Medicine, Barts and The London School of Medicine and Dentistry, Queen Mary University of London, London, United Kingdom; McGill University, Canada

## Abstract

**Background:**

There is little consensus, and minimal evidence, regarding the age at which to stop cervical screening. We studied the association between screening at age 50–64 y and cervical cancer at age 65–83 y.

**Methods and Findings:**

Cases were women (*n* = 1,341) diagnosed with cervical cancer at age 65–83 y between 1 April 2007 and 31 March 2012 in England and Wales; age-matched controls (*n* = 2,646) were randomly selected from population registers. Screening details from 1988 onwards were extracted from national databases. We calculated the odds ratios (OR) for different screening histories and subsequent cervical cancer. Women with adequate negative screening at age 65 y (288 cases, 1,395 controls) were at lowest risk of cervical cancer (20-y risk: 8 cancers per 10,000 women) compared with those (532 cases, 429 controls) not screened at age 50–64 y (20-y risk: 49 cancers per 10,000 women, with OR = 0.16, 95% CI 0.13–0.19). ORs depended on the age mix of women because of the weakening association with time since last screen: OR = 0.11, 95% CI 0.08–0.14 at 2.5 to 7.5 y since last screen; OR = 0.27, 95% CI 0.20–0.36 at 12.5 to 17.5 y since last screen. Screening at least every 5.5 y between the ages 50 and 64 y was associated with a 75% lower risk of cervical cancer between the ages 65 and 79 y (OR = 0.25, 95% CI 0.21–0.30), and the attributable risk was such that in the absence of screening, cervical cancer rates in women aged 65+ would have been 2.4 (95% CI 2.1–2.7) times higher. In women aged 80–83 y the association was weaker (OR = 0.49, 95% CI 0.28–0.83) than in those aged 65–69 y (OR = 0.12, 95% CI 0.09–0.17). This study was limited by an absence of data on confounding factors; additionally, findings based on cytology may not generalise to human papillomavirus testing.

**Conclusions:**

Women with adequate negative screening at age 50–64 y had one-sixth of the risk of cervical cancer at age 65–83 y compared with women who were not screened. Stopping screening between ages 60 and 69 y in women with adequate negative screening seems sensible, but further screening may be justifiable as life expectancy increases.

*Please see later in the article for the Editors' Summary*

## Abstract 2

Peter Sasieni and colleagues use a population-based case control study to assess the risk of cervical cancer in screened women aged over 65 years to help inform policy on the upper age of cervical cancer screening.

*Please see later in the article for the Editors' Summary*

## Editors' Summary

**Background:**

Nearly one in ten cancers diagnosed in women occur in the cervix, the structure that connects the womb to the vagina. Every year, more than a quarter of a million women (mostly in developing countries) die because of cervical cancer, which occurs only after the cervix has been infected with human papillomavirus (HPV) through sexual intercourse. In the earliest stages of cervical cancer, abnormal cells begin to grow in the cervix. Cells with low-grade abnormalities (changes that often revert to normal), cells with high-grade abnormalities (which are more likely to become cancerous), and cancer cells can all be detected by collecting a few cells from the cervix and examining them under a microscope. This test forms the basis of cervical screening, which has greatly reduced cervical cancer deaths in countries with a national screening program by ensuring that cervical abnormalities are detected at an early, treatable stage. In the UK, for example, since the start of a cervical screening program in 1988 in which women aged 25–64 years are called for testing every 3–5 years, the incidence of cervical cancer (the number of new cases per year) has almost halved at a time when sexually transmitted diseases have more than doubled.

**Why Was This Study Done?:**

Currently, there is little consensus about the age at which cervical screening should stop, and minimal evidence about the impact of cervical screening on the incidence of cervical cancer in older women. In this population-based case control study (a study that compares the characteristics of all the cases of a disease in a population with the characteristics of matched individuals without the disease), the researchers examine the association between screening in women aged 50–64 years and cervical cancer in women aged 65–83 years. They ask whether well-screened women with a history of negative results and no evidence of high-grade abnormalities are at sufficiently low risk of cervical cancer that screening can be stopped at age 65 years, and whether women who are regularly screened (at least once every 5.5 years) between the ages of 50 and 64 years are subsequently at reduced risk of cervical cancer.

**What Did the Researchers Do and Find?:**

The researchers randomly selected two age-matched controls for every woman aged 65–83 years who was diagnosed with cervical cancer between 2007 and 2012 in England and Wales. The researchers included 1,341 women with cervical cancer and 2,646 controls. They extracted each woman's cervical screening details from national databases and calculated the association between screening history and subsequent cervical cancer. Women with adequate negative screening at age 65 years (at least three tests at age 50–64 years with the last one over age 60, the last three of which were negative, and no evidence of high-grade abnormalities) were at the lowest risk of cervical cancer (20-year risk of eight cancers per 10,000 women) compared with unscreened women (20-year risk of 49 cancers per 10,000 women). That is, women who were not screened at age 50–64 years were six times more likely to develop cervical cancer between the ages of 65 and 83 years than women who were screened. The risk of developing cervical cancer among adequately negatively screened women increased with age and with time since the last screen. Finally, the researchers estimate that in the absence of any cervical screening, the rate of cervical cancer among women aged 65–79 years would be 23 cases per 100,000 woman-years, 2.4 times higher than the current rate.

**What Do These Findings Mean?:**

These findings show that women who exited the screening program in England and Wales with a history of adequate negative screening between the ages of 50 and 64 years were at a very low risk of being diagnosed with cervical cancer at the age of 65 years or older. The “protection” provided by screening was greatest for women aged 65–69 years and decreased steadily with increasing age and with time since the last negative screen. Because the researchers did not have any information on other characteristics that might have affected cervical cancer risk (for example, number of sexual partners), the women who were screened may have shared other characteristics that reduced their risk of developing cervical cancer. Moreover, these findings, which are based on microscopic examination of cells, may not generalise to the HPV-based screening programs that many countries are considering. Despite these limitations, the researchers conclude that, for now, it seems sensible to continue screening at least until age 60 years and not beyond age 69 years in women with adequate negative screening, but that given increasing life expectancy, screening in older women might be justified in the future.

**Additional Information:**

Please access these websites via the online version of this summary at http://dx.doi.org/10.1371/journal.pmed.1001585.

- This study is further discussed in a *PLOS Medicine* Perspective by Anne Rositch and colleagues
- The US National Cancer Institute provides information about cervical cancer for patients and for health professionals, including information on cervical screening (in English and Spanish)
- The US Centers for Disease Control and Prevention also has information about cervical cancer and about cervical screening
- The UK National Health Service Cervical Screening Programme website has detailed information and statistics on cervical screening in England
- The UK National Health Service Choices website has pages on cervical cancer (including a personal story about cervical cancer) and on cervical screening (including personal comments about screening)
- Cancer Research UK provides detailed information about all aspects of cervical cancer
- More information about cervical cancer and screening is available from the Macmillan cancer charity
- MedlinePlus provides links to additional resources about cervical cancer and screening (in English and Spanish)
- Personal stories about cervical cancer and about cervical screening are available through the charity Healthtalkonline

## Introduction

There is a lack of consensus regarding the appropriate upper age for cervical screening and little direct evidence on which to base policy [1],[2]. Until recently recommendations from the US have been for women to be screened into their 80 s, and cervical screening over age 65 y is common [3],[4]. The current recommendation from both the American Congress of Obstetricians and Gynecologists and the American Cancer Society is that screening should be stopped at age 65 y for women with evidence of adequate prior negative screening and no history of cervical intraepithelial neoplasia grade 2+ [5],[6], but this recommendation was based only on expert opinion and modelling because of the lack of empirical data. More radically, in 1993, Van Wijngaarden and Duncan [7] proposed that screening over the age of 50 y was of little value in those previously well screened. However Rebolj et al. [8] disagreed with these findings and reported a similar 10-y cumulative incidence of cervical cancer in women whose third consecutive negative test was taken at age 45–54 y and in those whose third test was at age 30–44 y. Internationally, the upper age for cervical screening varies from 59 or 60 y in Denmark, Finland, and Scotland, to 70 y and over in Japan, Korea, and Uruguay [9]. In England and Wales women receive their last test between the ages of 60 and 64 y. The justification for stopping screening in older women is based on the natural history of cervical cancer. Incident human papillomavirus (HPV) infections in women aged 55 y or over are rare and are unlikely to have sufficient time to progress to invasive cancer in the woman's lifetime. However, we know of no direct evidence of the impact of cervical screening in older women in the period 7 to 15 y after their last screening test.

We studied the association between screening women at age 50–64 y and cervical cancer diagnosed at age 65–83 y. Our aim was to provide policy-makers with evidence to help address the following questions. (1) Are well-screened women with a history of negative tests and no high-grade results at sufficiently low risk of cervical cancer that screening can cease at age 65? If so, how low is their risk, and how does it change as they age? (2) Are women who regularly participate in screening at age 50–64 y at reduced risk of cervical cancer at age 65–83 y?

## Methods

### Ethics Statement

The collection of data as part of this audit has been approved since 2003 (PIAG 1-08(a)/2003) under Section 251 of the National Health Service Act 2006, which re-enacted Section 60 of the Health and Social Care Act 2001. The analysis of anonymised data in this context is considered service evaluation and therefore according to UK guidelines is considered ethical without further consideration by a research ethics committee [10].

### Participants

There is free universal health care in England and Wales provided by the National Health Service (NHS), and all adult females registered with the NHS have a record in the national Cervical Screening Call/Recall System. Cases were women aged 65 y or older who were diagnosed with cervical cancer (ICD-10 C53) in England (between 1 April 2007 and 31 March 2012) and Wales (between 1 January 2007 and 31 December 2009) (October 2012 dataset) and who were registered with an NHS general practitioner (GP). Any other woman registered with an NHS GP at the time of a case diagnosis was eligible as a control for that case. Controls were randomly selected (using a computer program) from women satisfying the matching criteria. Two controls were individually matched to each case based on age and place of residence: one control had the same GP as the case, and a second control had a different GP but was within the same administrative area. Occasionally, only one control could be identified. The study design did not allow for collection of data on potential confounders such as sexual behaviour, parity, and smoking. Matching on the same GP was to provide a crude surrogate for socio-economic status and ethnicity. The reason for selecting a control from a different GP was to avoid overmatching if screening uptake was dependent on the GP's enthusiasm for cervical screening. Data were collected on all selected controls, so there was no study selection or participation bias.

Data on screening histories were abstracted from routinely recorded cervical cytology records held on the Cervical Screening Call/Recall System (and as such were not subject to recall bias). These records include all NHS (and many private provider) smears taken in the UK since 1988.

After local NHS staff linked screening data to cases and controls, the data were anonymised locally before being transferred for cleaning and analysis. Guidelines on the collection of data for this audit and details of the design have been published previously [11]–[13].

Delays in the inclusion of newly diagnosed cancers in our audit resulted in advanced stage cancers and cases diagnosed in the most recent period being underrepresented. It was estimated that the database contained 78% of all cancers in women aged 65–83 y diagnosed during the study period in England [14].

Women aged 60 y or over on 1 January 1988 were excluded because they may not have been invited for screening as part of the Cervical Screening Call/Recall System; therefore, relatively few women in the study were diagnosed with cancer over the age of 80.

Cytology results were classified according to the British Society for Clinical Cytology system [15]. The British Society for Clinical Cytology terminology can be broadly compared to the Bethesda System, as follows: “borderline changes” include atypical squamous cells, atypical glandular cells, and “borderline, high-grade dyskaryosis not excluded” (equivalent to the Bethesda System's ASC-H); “mild dyskaryosis” corresponds to low-grade squamous intraepithelial lesion (LSIL); “moderate and severe dyskaryosis” corresponds to high-grade squamous intraepithelial lesion (HSIL); and there are separate categories, “query invasive” and “query glandular neoplasia”, for squamous cell carcinoma and adenocarcinoma in situ/cervical glandular intraepithelial neoplasia/adenocarcinoma, respectively. Tests classified as “inadequate” should have resulted in immediate repeat testing and were therefore ignored in this analysis. The exception was when they resulted in a referral to colposcopy (guidelines recommended referral after a third consecutive inadequate test).

### Statistical Analysis

We used conditional logistic regression to estimate the odds ratio (OR) of cervical cancer (at age 65 y or older) for women with various screening patterns compared to those with no cervical cytology (except possibly inadequate test[s] not resulting in referral) between the ages 50 and 64 y. To exclude screen-detected cancers (diagnosed following screening at age 64 y), we excluded women (including control women) diagnosed at age 65.0–65.5 y with a cytology test within 6 mo of case diagnosis. Age of diagnosis was defined for controls using the date of diagnosis for their matched case.

We calculated absolute risks using a weighted logistic regression model, with weights calculated by case status, age category (65–69, 70–74, 75–79, and 80–84 y), and year of diagnosis. Weights for cases were calculated as the total number of cases diagnosed (according to the official cancer registration statistics for England [MB1 series] [16] [age-specific data for 1975–2011 were prepared by Cancer Research UK] and for Wales [17]) divided by the number recorded in the audit (see Table S1). As official figures have not yet been published for 2012, the 2011 weights were used. For controls, for each year of diagnosis and age group, the weights were calculated using the following formula:
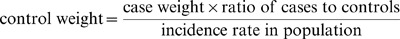
(1)Cervical cancer incidence rates were calculated as the number of cases diagnosed according to the MB1 series divided by the mid-year population estimate. We used the ORs and the percentage of cases in each category to calculate the population attributable risk (under the assumption of the association being causal) in two ways. We calculated how much greater cervical cancer rates (in women aged 65–79 y) would have been in the absence of screening beyond age 50. Additionally, we calculated the proportion of cancers (that did occur) that might have been prevented had all women been screened at least every 5.5 y between the ages 50 and 64 y. Confidence intervals for the population attributable risks were calculated by bootstrapping (percentile method based on 2,000 replications).

We studied the association between cervical screening at age 50–64 y and the risk of cervical cancer at age 65–83 y by answering the following questions. (1) What is the risk of cervical cancer at age 65 y and older in women with a history of negative tests and no high-grade results at age 50–64 y? Does the risk relative to women not screened at age 50–64 y change with time since last test (i.e., as women age)? (2) What is the risk of cervical cancer at age 65–83 y in women who regularly participate (defined as having a test at least every 5.5 y) in screening at age 50–64 y compared with the risk in women not screened at all at age 50–64 y?

To address the first question, screening histories for women between the ages of 50 and 64 y were divided into four categories: (1) “adequate negative screening”, defined as women with at least three tests at age 50–64 y (with at least one at age 60–64 y), the last three of which were negative, and no HSIL or worse cytology since age 50; (2) “sub-optimal but negative screening”, defined as women not satisfying “adequate negative screening” but with either at least one negative test and no abnormal tests, or with three consecutive negative tests and no HSIL, but the last test was under age 60; (3) “abnormal screening”, defined as women with HSIL cytology at age 50–64 y, regardless of whether or not it was followed by three consecutive negatives, or with a low-grade result (atypical squamous cells of undetermined significance [ASC-US] or LSIL) that was not followed by three consecutive negatives; (4) “no screening”, defined as women with no test at age 50–64 y (excluding inadequate tests not resulting in referral to colposcopy). The first category approximates the group that the American Congress of Obstetricians and Gynecologists would discharge from screening. As we have no access to histology records of cervical intraepithelial neoplasia, we cannot identify those with cervical intraepithelial neoplasia grade 2+ and instead exclude women with HSIL cytology at any time since age 50.

Attenuation of the OR with increasing age was investigated non-parametrically (ORs were calculated separately for individuals diagnosed at different ages using overlapping 4-y intervals every 6 mo from age 65.0–69.0 y to age 79.0–83.0 y) and parametrically (a trend line and 95% confidence interval were calculated by including an interaction term between negative screening and age at diagnosis in the regression model). We also studied the risk of cervical cancer in women with at least three consecutive negative tests and no HSIL cytology at age 50–64 y by time since last test (at age 50–64 y).

To address the second question (if screening per se is associated with a lower risk of cervical cancer), a woman's (maximum) screening interval was defined as the longest period during the 15 y from age 50 to age 64 y in which she did not have an adequate cytology test (for women who were over 50 y in 1988, we considered only the interval from 1 January 1988 until their 65th birthday). In England and Wales, women in this age group were invited either every 3 y or every 5 y (depending on local policy at the time); therefore, we defined women whose maximum interval between tests during the 15 y period under study was at most 5.5 y to have attended screening regularly. Women with only inadequate tests were considered not to have been screened at age 50–64 y unless referred to colposcopy as a result of the inadequate cytology, in which case they were considered to be in category 3, “abnormal screening”.

The main analyses were repeated in a number of subgroups (e.g., by histological type) and with a number of exclusions (e.g., considering only cancers known to be stage 1B or worse). Additionally, we performed a sensitivity analysis of the possible impact of unknown confounders (such as smoking or number of sexual partners) on our results, and estimated the potential impact of changing the age at last screen on cervical cancer rates. We considered a risk score of the sort obtained from questionnaire data (not including screening attendance), divided into five risk levels, with 10% of screened women in each of the extreme groups, 20% in each of the high- and low-risk groups, and 40% in the middle group. We allowed for a 5-fold difference in risk between the highest and lowest risk groups, a difference in risk we believe to be plausible but extreme [18]. We assumed that the distribution of risk levels in unscreened women is a logistic shift of the distribution in screened women (shifted by −ln[2.25]) corresponding to an OR of 4.25 between the extreme groups.

Analyses were done in STATA 12 (StataCorp).

## Results

A total of 1,341 women with invasive cervical cancer diagnosed at age 65–83 y and 2,646 matched controls were included in the study. Thirty-six cases (2.7%) had only one control—either because, for example, the other potential control was born before 1928 and the case was not, or because only one control that fulfilled the matching criteria was found. The distribution of cases by age, year of diagnosis, International Federation of Gynecology and Obstetrics (FIGO) stage, and histology is shown in Table 1. Similar numbers of women (404–435) were diagnosed in each 5-y age group for 65–79 y, but only 97 women were aged 80–83 y. Most of the cancers in this study were diagnosed as FIGO stage 2 or worse, and over 70% were squamous cell carcinoma.

**Table 1:** Number and percent of invasive cervical cancer cases by age, year of diagnosis, FIGO stage, and histology.

Characteristic	*n*	Percent
**Age at Diagnosis**		
65–69 y	435	32.4%
70–74 y	404	30.1%
75–79 y	405	30.2%
80–83 y	97	7.2%
**Year of diagnosis**		
2007	212	15.8%
2008	285	21.3%
2009	289	21.6%
2010	247	18.4%
2011	263	19.6%
2012	45	3.4%
**FIGO stage**		
1A	63	4.7%
1B	267	19.9%
2+	696	51.9%
Unknown	315	23.5%
**Histology**		
Squamous cell carcinoma	940	70.1%
Adenocarcinoma	238	17.7%
Other	101	7.5%
Unknown	62	4.6%
**Total**	1,341	100%

Table 2 shows the risk of cervical cancer diagnosed at age 65–83 y by screening history in the age interval 50–64 y. The highest risk was observed in those with a history of abnormal cytology (OR = 1.83, 95% CI 1.37–2.43, when compared with women without any tests at age 50–64 y). Women with cervical cancer were more likely to have had no screening at age 50–64 y than the general population (40% versus 16%) and less likely to have had adequate negative screening (21% versus 53%). Women with adequate negative screening were approximately six times less likely to be diagnosed with cervical cancer at age 65 y or older (OR 0.16, 95% CI 0.13–0.19) compared with women with no (adequate) tests since age 50.

**Table 2:** Risk of cervical cancer at age 65–83 y by screening history at age 50–64 y.

Screening History at Age 50–64 y^a^	Cases		Controls		OR with No Screening as Reference		OR with Adequate Negative Screening as Reference		Absolute Risk (per 100,000 Women per Year)	20-y Risk (per 1,000 Women)
	*n*	Percent	*n*	Percent	OR	95% CI	OR	95% CI		
Adequate negative	288	21.5	1,395	52.7	0.16	0.13–0.19	1	Reference	4.0	0.8
Sub-optimal but negative	300	22.4	724	27.4	0.34	0.28–0.42	2.15	1.76–2.64	8.7	1.7
Abnormal	221	16.5	98	3.7	1.83	1.37–2.43	11.52	8.57–15.47	43.0	8.6
No screening	532	39.7	429	16.2	1	Reference	6.31	5.17–7.69	24.5	4.9

^a^ Adequate negative: last three tests were negative (at least one at age 60–64 y) and no high-grade (HSIL) or worse cytology since age 50. Sub-optimal but negative: not satisfying “adequate negative” but with either at least one negative test and no abnormal tests, or with three consecutive negatives and no HSIL but with the last test before age 60. Abnormal: HSIL cytology or a low-grade result (ASC-US or LSIL) not followed by three negatives. No screening: no test at age 50–64 y.

**Figure 1:**
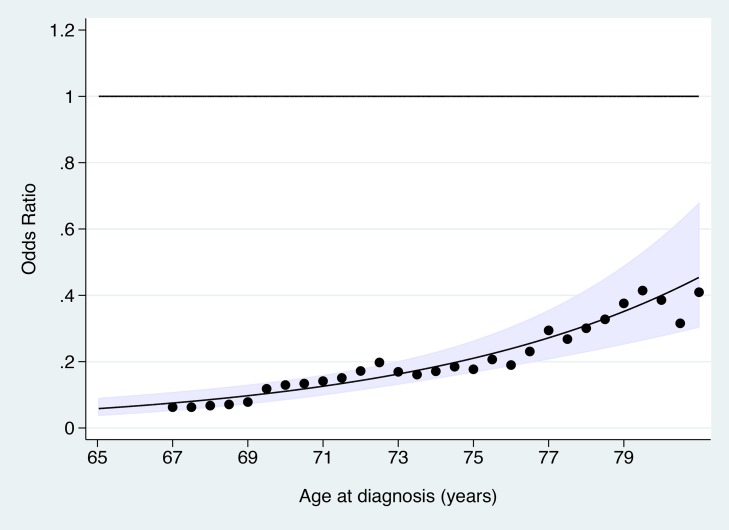
Odds ratio of cervical cancer at age 65–83 y in those with adequate negative screening compared with no screening at age 50–64 y by age at diagnosis. The line shows the log-linear trend, the shaded area shows the 95% confidence interval for the trend line, and the dots provide estimates based on data within 2 y of the *x*-axis values.

**Figure 2:**
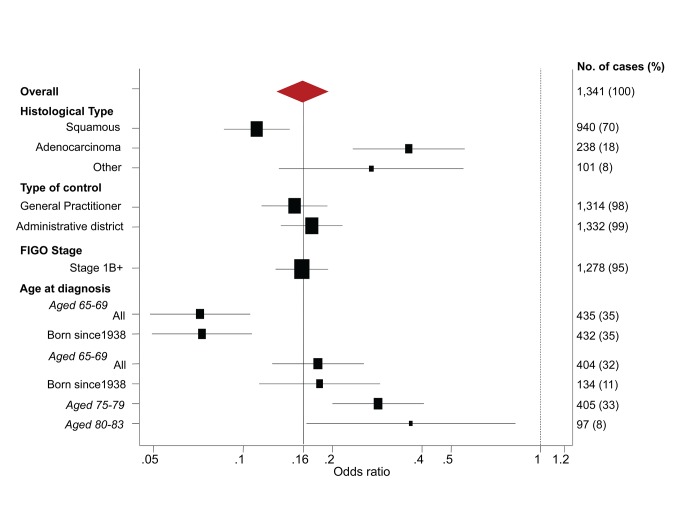
Subgroup analyses—odds ratios of cervical cancer at age 65–83 y for women with adequate negative screening relative to no screening at age 50–64 y.

**Figure 3:**
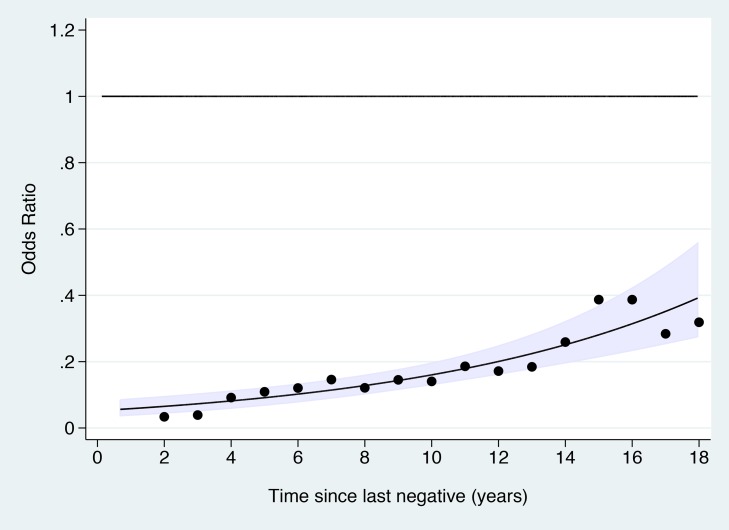
Odds ratio of cervical cancer in those with adequate negative screening compared with no screening at age 50–64 y by time since last screen. The line shows the log-linear trend, the shaded area shows the 95% confidence interval for the trend line, and the dots provide estimates based on data within 2 y of the *x*-axis values.

The OR for cervical cancer in the adequate negative screening group increased with age (Figure 1), from 0.07 (95% CI 0.05–0.11) for ages 65–69 y to 0.28 (95% CI 0.20–0.41) for ages 75–79 y and to 0.37 (95% CI 0.16–0.82) for ages 80–83 y (Figure 2; Table 3). Similarly, in women whose last three tests were negative and who had no HSIL results at age 50–64 y, the low risk associated with a negative screen weakened with time since last screen (Figure 3). The OR was 0.11 (95% CI 0.08–0.14) for women diagnosed within 2.5 to 7.5 y of their last screen, but the OR was 0.27 (95% CI 0.20–0.36) for those diagnosed 12.5 to 17.5 y after the last screen. There was no evidence that this tapering effect depended on the age at last screen, but we had little power to address this issue, as 80% of women with three negatives had their last test between 60.9 and 64.6 y of age.

**Table 3:** Risk of cervical cancer according to screening history at age 50–64 y by age at diagnosis.

Screening History	Cases (*n*)	Controls (*n*)	OR	95% CI
**Age at diagnosis 65–69 y**				
Adequate negative	73	539	0.07	0.05–0.11
Sub-optimal but negative	66	166	0.22	0.15–0.33
Abnormal	83	46	0.92	0.58–1.47
No screening	213	108	1	Reference
**Age at diagnosis 70–74 y**				
Adequate negative	115	488	0.18	0.13–0.25
Sub-optimal but negative	78	174	0.38	0.25–0.56
Abnormal	72	28	2.20	1.31–3.70
No screening	139	117	1	Reference
**Age at diagnosis 75–79 y**				
Adequate negative	86	326	0.28	0.20–0.41
Sub-optimal but negative	121	294	0.45	0.33–0.63
Abnormal	56	21	3.04	1.67–5.54
No screening	142	157	1	Reference
**Age at diagnosis 80–83 y**				
Adequate negative	14	42	0.37	0.16–0.82
Sub-optimal but negative	35	90	0.46	0.25–0.82
Abnormal	10	3	5.34	1.12–25.46
No screening	38	47	1	Reference

Based on cervical cancer incidence in women aged 65–84 y in 2007–2011, we estimated the absolute rate per 100,000 woman-years to be 4.0 in adequately negatively screened women, 24.5 in unscreened women, and 43.0 in women with an abnormal screening history. These absolute risks translate to a 20-y risk per 10,000 women of eight for adequately negatively screened women and 86 for those with abnormal screening.

The OR of squamous cell carcinoma (Table 4; Figure 2) associated with adequate negative screening was lower than for all cervical cancer (OR = 0.11, 95% CI 0.09–0.14), whereas the OR for adenocarcinoma of the cervix was greater (OR = 0.36, 95% CI 0.23–0.56).

**Table 4:** Risk of cervical cancer at age 65–83 y by screening history at age 50–64 y and histological type.

Screening History at Age 50–64 y^a^	Cases		Controls		OR	95% CI
	*n*	Percent	*n*	Percent		
**Squamous cell carcinoma**						
Adequate negative	149	15.9	979	52.7	0.11	0.09–0.14
Sub-optimal but negative	202	21.5	502	27.0	0.31	0.25–0.40
Abnormal	179	19.0	75	4.0	1.80	1.29–2.53
No screening	410	43.6	300	16.2	1	Reference
**Adenocarcinoma**						
Adequate negative	79	33.2	241	51.3	0.36	0.23–0.56
Sub-optimal but negative	61	25.6	136	28.9	0.51	0.32–0.82
Abnormal	30	12.6	15	3.2	2.07	1.05–4.10
No screening	68	28.6	78	16.6	1	Reference
**Other**						
Adequate negative	41	40.6	114	57.6	0.27	0.13–0.55
Sub-optimal but negative	23	22.8	49	24.7	0.38	0.18–0.82
Abnormal	6	5.9	7	3.5	0.70	0.19–2.46
No screening	31	30.7	28	14.1	1	Reference

^a^ Adequate negative: last three tests were negative (at least one at age 60–64 y) and no high-grade (HSIL) or worse cytology since age 50. Sub-optimal but negative: not satisfying “adequate negative” but with either at least one negative test and no abnormal tests, or with three consecutive negatives and no HSIL but with the last test before age 60. Abnormal: HSIL cytology or a low-grade result (ASC-US or LSIL) not followed by three negatives. No screening: no test at age 50–64 y.

**Figure 4:**
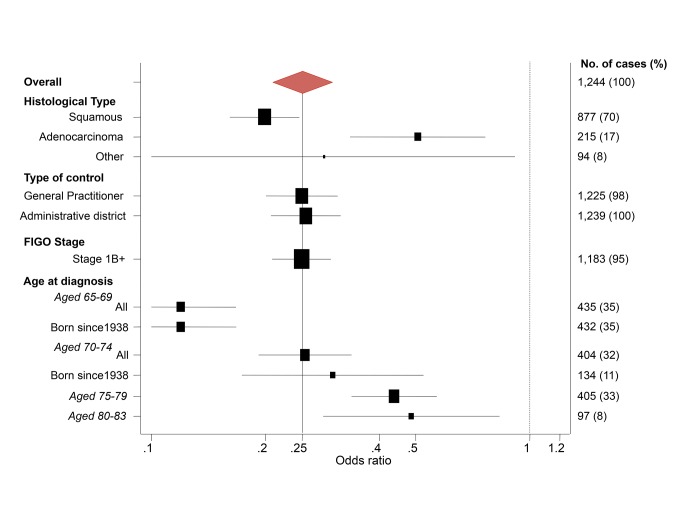
Subgroup analyses—odds ratios of cervical cancer at age 65–79 y for women screened at least every 5.5 y at age 50–64 y relative to no screening at age 50–64 y.

To explore the effect of screening per se (as opposed to the association with negative screening), we estimated the association (OR) between a diagnosis of cervical cancer at age 65–83 y and the maximum screening interval at ages 50–64 y (Table 5). The lowest risk was seen for those with a screening interval of ≤5.5 y (OR = 0.25, 95% CI 0.21–0.30, compared with those with no screen recorded at age 50–64 y): screening intervals of ≤3.5 y were no more “protective” than those of 3.5–5.5 y. Even women with a screening interval of 9–15 y had significantly lower risk than those not screened at all at age 50–64 y (OR = 0.54, 95% CI 0.40–0.71). The lower risk associated with the currently recommended screening every 5 y (interval ≤5.5 y) at ages 50–64 y diminished with increasing age at diagnosis: 65–69 y, OR = 0.12 (95% CI 0.09–0.17); 70–74 y, OR = 0.27 (95% CI 0.19–0.36); 75–79 y, OR = 0.46 (95% CI 0.35–0.61); and 80–83 y, OR = 0.49 (95% CI 0.28–0.83) (Figure 4; Table 6).

**Table 5:** Risk of cervical cancer at age 65–79 y by maximum screening interval between the ages 50 and 64 y.

Screening Interval at Age 50–64 y	Cases		Controls		OR	95% CI
	*n*	Percent	*n*	Percent		
Not screened at age 50–64 y	510	41.0	411	16.7	1	Reference
≤3.5 y	149	12.0	464	18.8	0.27	0.21–0.34
3.5–5.5 y	326	26.2	1,060	43.0	0.25	0.20–0.30
5.5–9 y	150	12.1	369	15.0	0.34	0.26–0.43
9–15 y	109	8.8	160	6.5	0.54	0.40–0.71
≤5.5 y	475	38.2	1,524	61.9	0.25	0.21–0.30

**Table 6:** Odds ratios of cervical cancer by maximum screening interval at age 50–64 y relative to no screening, by age at diagnosis.

Screening Interval	Diagnosed at Age 65–69 y				Diagnosed at Age 70–74 y				Diagnosed at Age 75–79 y				Diagnosed at Age 80–83 y			
	Case *n*	Control *n*	OR	95% CI	Case *n*	Control *n*	OR	95% CI	Case *n*	Control *n*	OR	95% CI	Case *n*	Control *n*	OR	95% CI
Not screened at age 50–64 y	213	108	1	Reference	148	127	1	Reference	149	176	1	Reference	42	54	1	Reference
≤3.5 y	26	158	0.09	0.05–0.15	47	144	0.28	0.19–0.43	76	162	0.56	0.39–0.81	24	74	0.37	0.19–0.70
3.5–5.5 y	90	360	0.13	0.09–0.19	109	353	0.26	0.18–0.36	127	347	0.42	0.30–0.57	27	52	0.65	0.34–1.22
5.5–9 y	46	155	0.16	0.10–0.24	57	123	0.42	0.28–0.62	47	91	0.59	0.39–0.90	3	2	2.26	0.37–13.85
9–15 y	60	78	0.40	0.26–0.62	43	60	0.59	0.36–0.94	6	22	0.30	0.12–0.78	1	0	—	—
≤5.5 y	116	518	0.12	0.09–0.17	156	497	0.27	0.19–0.36	203	509	0.46	0.35–0.61	51	126	0.49	0.28–0.83

The estimated ORs depended on the age at diagnosis and histological type, but were otherwise very similar in different subgroups (Figures 2 and 4). Restricting analysis to cases known to have stage 1B or worse cancer or to cases born since 1938 gave (age-specific) ORs that were extremely similar to those without such restrictions. Similarly, the results were extremely similar using either only the GP control or only the district control.

Treating the associations in Table 5 as causal, we estimated that in the absence of screening at ages 50–64 y, cervical cancer rates in women aged 65–79 y (currently 9.6 per 100,000 woman-years) [19] would have been 2.42 (95% CI 2.11–2.71) times higher (i.e., 23 per 100,000 woman-years). Conversely, had all women been screened at intervals of ≤5.5 y between the ages 50 and 64 y, population rates of cervical cancer in women aged 65–79 would have been 38% (95% CI 32%–42%) lower than those observed (corresponding to a rate of 5.9 per 100,000 woman-years).

It is possible that the distribution of risk factors (other than cervical screening) for cervical cancer differed between screened and unscreened women. Taking what we consider to be a plausible but extreme scenario (Table 7), resulted in an 18% ( = 1/0.85−1) increase in the estimated ORs, i.e., the ORs of cervical cancer in regularly screened women compared with never screened women would be 0.14 for women aged 65–69 y, 0.32 for women aged 70–74 y, and 0.54 for women aged 75–79 y.

**Table 7:** Estimated relative risks of cervical cancer associated with questionnaire-type risk factor data (e.g., economic deprivation, number of sexual partners, and smoking).

Statistic	Risk Level					Weighted Average Risk	Relative Risk
	Very Low	Low	Medium	High	Very High		
Relative risk	0.4	0.7	1.0	1.4	2.0		
Screened (percent with risk factor)	10.0	20.0	40.0	20.0	10.0	1.06	0.85
Not screened (percent with risk factor)	4.7	11.3	34.9	29.1	20.0	1.25	

For illustration of what might be the effect of changing the age at last screen, we have estimated the potential impact of stopping screening at age 65 y (as per our data), age 55, or age 75 y (Table 8). In 1975 the cumulative incidence was 892 cervical cancers per 100,000 women. With screening until age 65, this value would be reduced to 211 using the ORs from this study as relative risks, and 250 with the adjustment for unobserved confounding. Using the observed relative risks, there would be an additional 182 cancers if cervical cancer screening ceased at age 55, and 103 fewer if it continued until age 75. Interestingly, the added benefit of prolonged screening is greater with the adjusted relative risks: 216 additional cervical cancers when stopping screening at age 55, and 122 fewer when continuing screening to age 75.

**Table 8:** Modelling of the effect of ceasing screening at age 55 or age 75(for unknown confounders) relative risks for women screened at least every 5.5 y up to age 65 y.

Characteristic	No Screening: Baseline Rates^a^	Screening until Age 65 y				Screening until Age 55 y				Screening until Age 75 y			
		Observed RR^b^	Rate in Those Screened	Adjusted RR^c^	Adjusted Rate in Those Screened	Relative Risks^d^	Rate in Those Screened	Adjusted RR^c^	Adjusted Rate in Those Screened	Relative Risks^d^	Rate in Those Screened	Adjusted RR^c^	Adjusted Rate in Those Screened
**Age**													
55–59 y	34.9	*0.08*	2.8	0.11	3.9	0.12	4.2	0.17	5.9	*0.08*	2.8	0.11	3.9
60–64 y	34.6	*0.08*	2.8	0.11	3.9	0.27	9.3	0.38	13.2	*0.08*	2.8	0.11	3.9
65–69 y	28.3	0.12	3.4	0.17	4.8	0.46	13.0	0.65	18.3	*0.08*	2.3	0.11	3.2
70–74 y	28.1	0.27	7.6	0.38	10.7	0.49	13.8	0.69	19.4	*0.08*	2.2	0.11	3.2
75–79 y	26.4	0.46	12.1	0.65	17.1	*0.65*	17.2	0.92	24.2	0.12	3.2	0.17	4.5
80–84 y	26.2	0.49	12.8	0.69	18.1	*0.70*	18.3	0.99	25.8	0.27	7.1	0.38	10.0
**Number of cancers at age 55–84 y per 100,000 women**	892		208		292		379		534		102		143
**Extra cancers compared to screening until 65 y**							171		241		−106		−149

^a^ Age-specific incidence rates (per 100,000) for England in 1975.

^b^ See Table 6 for age-specific relative risks, with the exception of those in italics, which are extrapolations.

^c^ The adjusted RR is the observed RR divided by the adjustment factor of 0.85 from Table 7.

^d^ Observed RRs are shifted by 10 y.

## Discussion

This study showed that women with adequate negative screening at age 50–64 y (women whose last three tests were negative [with at least one at age 60–64 y] and who had no high-grade cytology between the ages of 50 and 64 y) were at particularly low risk of being diagnosed with cervical cancer at age 65 y or older: the risk is 84% less than in unscreened women. The 20-y absolute risk of cervical cancer was eight per 10,000 women in those so screened compared to 49 per 10,000 women in those not screened between the ages of 50 and 64 y. Similarly, low risk of cervical cancer was observed among women whose interval between tests at ages 50–64 y was no greater than 5.5 y. The “protection” of adequate negative screening at age 50–64 y was greater for women aged 65–69 y and decreased steadily with time since last negative screen. It was considerably less 15 y after screening than 10 y after screening. Similarly, regular screening (interval ≤5.5 y) at ages 50–64 y was associated with a low risk of cervical cancer until age 75; thereafter, the “effect” of screening weakened, and by age 80 y the risk in well-screened women was about half the risk of unscreened women. These results were robust to a number of sensitivity analyses.

This is the largest study looking at cervical screening and the risk of cervical cancer at age 65 y and older. Controls were automatically selected from the database of all women invited for cervical screening in England and Wales, minimising the selection bias, and linkage was used to obtain all screening histories, ensuring completeness of the data and no recall bias. The design (a population-based case control study) allowed estimation of absolute risks.

The main limitation of this observational study is that the database containing screening histories (from which the controls were identified) did not record information on risk factors. The association with screening in this study is greater for squamous cell carcinoma than for adenocarcinoma. The risk factors for both histological types are similar [18], and the vast majority of in situ lesions detected by screening are squamous cell carcinomas [20]. Together these three facts suggest that the greater association with squamous cell carcinoma is due to screening and not confounding. Additionally, the association was greater within 5 y (compared with 12.5–17.5 y) of screening, and we see no reason why the impact of unrecorded risk factors would change with time from last screen. Note that screening was not offered and was extremely rare after the age of 64 y, and coverage in women aged 50–64 y has been stable at about 80% over the last 16 y [20],[21]. Nevertheless, the underlying risk of cervical cancer in a woman who has never been screened may be greater than in a woman who is screened regularly. However, the only study that we could find of screening and cervical cancer that adjusts for risk factors (smoking) found that the adjusted and unadjusted ORs were similar [22]. Further, a study comparing never-attenders at cervical screening to attenders in Denmark found that never-attenders had no overrepresentation of cancer risk factors [23]. A small number of women in our cohort may have been screened before 1988 but not since, and we would not know about such early screening. However, analyses restricted to women who were under age 50 y in 1988 did not substantially change the age-specific effects of screening. Another limitation of our study is that we had only 97 cases over age 80 y and none over age 83 y, so estimates beyond age 80 y have wider confidence intervals, and we are unable to say what happens 20 y after the last screen taken at age 60–64 y (i.e., in women adequately negatively screened).

There has been only limited evidence and a lack of consensus regarding the optimal upper limit for screening. Even at younger ages there has been little study of the risk of cervical cancer more than 6 y after the last screening test. A smaller study by Kamineni et al. [22] including 69 cases in women aged 55–79 y (with a maximum time since last negative test of 7 y) also found a reduction in risk of developing cervical cancer within 5 y of the last negative test.

Our results do not give cause for concern regarding the current US recommendations to cease cervical screening in previously well-screened women at age 65 y. This recommendation has been supported by recently published data from a study in northern California [24]. However, while screening at age 50–64 y offers some protection long term, the magnitude of the protection decreases with time.

Screening in older women can be uncomfortable, and a lack of oestrogen can make obtaining an adequate cytology sample and interpreting it difficult. Although HPV testing on a sample collected without a speculum would overcome these problems for women testing HPV negative, those testing positive would still (currently) require cytology triage, and some would require colposcopy. Further, those women who test positive for HPV but who have normal cytology (approximately 4% [25]) pose a challenge for clinical management. Indeed, in the context of primary HPV screening, there will be the need for ongoing reassessment about the optimal age of stopping screening in relation to screening history.

The absolute risks in this paper are based on current rates of cervical cancer in older women in England. The absolute impact in the UK may be greater over the next 15 y because of the increasing underlying risk in cohorts born since 1950 [26]–[28]. Applying the same relative risks to a population with an underlying age-specific annual rate of cervical cancer of about 80 per 100,000 women between the ages 65 and 79 y, for every 1,000 women screened regularly between the ages 50 and 64 y, there would be approximately nine fewer cancers between the ages of 65 and 79 y.

Taken with the considerable evidence that cervical screening causally reduces cervical cancer incidence [29], the results here suggest that screening women aged 50–64 y substantially reduces their risk of cervical cancer at age 65 y and older, but that the magnitude of that protection decreases with time since last screen. The low risk of cervical cancer in those with a test at least every 5.5 y from age 50 to age 64 y (OR = 0.25) can to a large extent be attributed, in the authors' view, to the protection offered by screening. Based on this result, we estimated the annual rate of cervical cancer in the absence of screening to be 25 per 100,000 women. Interestingly this rate is slightly lower than the rate in women aged 65–79 y in England (27.6 per 100,000) before the screening programme was introduced (i.e., 1975–1988) [16].

The lower OR associated with adequate negative screening (0.16) than with regular screening (0.25) represents the ability of screening to identify those at particularly low risk of developing cancer in the following few years. This finding suggests that the guidelines recommending that women should exit screening only if their last three tests were all normal (and they satisfy the criteria for being adequately negatively screened) are sensible. Similarly, the high risk of cervical cancer (20-y absolute risk of 0.86%) in women with an unresolved abnormal test result in their history when they reach age 65 y emphasises the need for continued surveillance for such women (at least until three consecutive negatives are obtained). Among those with adequate negative screening, ceasing screening at age 50 y is unlikely to provide lifelong protection, but continuing screening till age 80 y is not necessary. As life expectancy increases (at age 65 y it is about 20 additional years for women in many industrialised countries [30]), consideration should be given to increasing the upper age of screening, possibly by extending the final screening interval from 5 to 10 y. This study provides evidence that should help produce cost-effectiveness analyses that would enable policy-makers to reach a rational decision.

Cervical screening is changing; most countries with organised screening have moved from conventional cytology to liquid-based cytology, and many are now considering primary HPV testing. This study was done within the UK with a mix of conventional cytology and liquid-based cytology, and as such, these results may not be generalisable to HPV-based screening programmes. Since the long-term negative predictive value of HPV testing is even better than that of cytology, one would expect the period of low risk to be longer following an HPV test, but there are currently no studies looking at the risk 15–20 y after a negative HPV test.

Cervical screening in women aged 50–64 y has a substantial impact on cervical cancer rates not only at age 50–64 y, but for many years thereafter. Screening up to age 65 y greatly reduces the risk of cervical cancer in the following decade, but the protection weakens with time and is substantially less 15 y after the last screen. In the light of increasing life expectancy, it would seem inappropriate for countries that currently stop screening between the ages 60 and 69 y to consider reducing the age at which screening ceases. To the contrary, consideration should be given to cost-effective ways of increasing the age of the last screening.

## Supporting Information

Checklist S1 **STROBE checklist.**
(DOC)

Table S1 **Calculation of cervical cancer incidence rate and weights for population controls.**
(XLSX)

## Abbreviations

ASC-US: atypical squamous cells of undetermined significance
FIGO: International Federation of Gynecology and Obstetrics
GP: general practitioner
HPV: human papillomavirus
HSIL: high-grade squamous intraepithelial lesion
LSIL: low-grade squamous intraepithelial lesion
NHS: National Health Service
OR: odds ratio
